# Comprehensive molecular epidemiology of BVDV in yaks (*Bos gruniens*) in Qinghai, China: high prevalence and dominance of BVDV-1u

**DOI:** 10.3389/fcimb.2025.1652023

**Published:** 2025-08-14

**Authors:** Zhi Li, Yuan Han, Yong Fu, Qing Yuan, Shuqin Wang, Xingye Pan, Wanchao Xue, Hong Yin, Shandian Gao, Ru Meng

**Affiliations:** ^1^ Qinghai Provincial Key Laboratory of Pathogen Diagnosis for Animal Diseases and Green Technical Research for Prevention and Control, Academy of Animal Sciences and Veterinary Medicine, Qinghai University, Xining, China; ^2^ Xining Animal Disease Prevention and Control Center, Xining, China; ^3^ Animal Husbandry and Veterinary Station of Huangyuan County, Xining, China; ^4^ Gansu Province Research Center for Basic Disciplines of Pathogen Biology, State Key Laboratory for Animal Disease Control and Prevention, Lanzhou Veterinary Research Institute, Chinese Academy of Agricultural Sciences, Lanzhou, China

**Keywords:** Bovine Viral Diarrhea Virus, yak (*Bos grunniens*), prevalence, phylogenetic analysis, subgenotypes

## Abstract

**Introduction:**

Bovine viral diarrhea virus (BVDV) is a major pathogen affecting livestock health in China. However, the current epidemiological status in yaks (*Bos grunniens*), particularly in Qinghai Province, remains insufficiently understood.

**Methods:**

In the present study, a comprehensive serological and molecular investigation of BVDV in yaks was conducted across broad geographic areas of eight administrative regions including Yushu, Guoluo, Huangnan, Hainan, Haidong, Haixi, Haibei, and Xining in Qinghai Province.

**Results:**

The results revealed widespread BVDV exposure in Qinghai yak, with an overall antibody prevalence of 84.52% (1158/1370) and substantial herd variation (12.00~98.07%). Active infections were confirmed through antigen detection, revealing prevalence ranging from 0.34% (Haixi) to 4.90% (Huangnan). Genetic characterization identified two circulating subgenotypes: BVDV-1a (n=3) and the predominant BVDV-1u (n=30), with the latter dominating across all regions.

**Discussion:**

These results highlight the endemic circulation of BVDV in Qinghai yak populations and uncover unexpected genetic diversity, emphasizing the need for control measures to mitigate the adverse impacts of BVDV infection in yaks in high-altitude pastoral systems.

## 1 Introduction

Bovine Viral Diarrhea (BVD), caused by the Bovine Viral Diarrhea Virus (BVDV), is classified as a notifiable infectious disease by the World Organization for Animal Health (WOAH). Cattle infected with BVDV may exhibit subclinical infection or symptoms, such as transient fever, diarrhea, leukopenia, respiratory signs and hemorrhagic syndrome, while transplacental infections in pregnant cattle can lead to embryonic death, abortions or the birth of persistently infected (PI) calves that are prone to mucosal disease (MD) (Baker, 1995). BVDV is a member of the *Flaviviridae* family, specifically within the *Pestivirus* genus and the viruses can be classified into several distinct genotypes, namely BVDV-1 (Pestivirus A), BVDV-2 (Pestivirus B), and BVDV-3/HoBi-like pestivirus (Pestivirus H) (Liu et al., 2009; Bauermann and Ridpath, 2015; Postel et al., 2021). Among these, BVDV-1 is widely prevalent worldwide with multiple proposed subgenotypes (1a to 1x), whereas BVDV-2 and BVDV-3 has comparatively limited subgenotypes (2a to 2e for BVDV-2 and HobiPeV a-e for BVDV-3) (Yeşilbağ et al., 2017; Deng et al., 2020; de Oliveira et al., 2022; Kalaiyarasu et al., 2022; Mucellini et al., 2023).

In recent years, BVDV has become a growing concern in cattle industry in China, posing substantial economic challenges. Systematic reviews and meta-analysis have provided robust epidemiological insights and demonstrated pooled BVDV prevalence of 53.0% and 36.0% in dairy cattle and yaks (*Bos gruniens*) respectively (Ran et al., 2019; Diao et al., 2020). The genetic diversity of BVDV in China has been characterized, revealing the presence of multiple subgenotypes, including 1a, 1b, 1c, 1d, 1m, 1o, 1p, 1q, 1u, 1v, 1w, 2a and 2b, as well as BVDV-3 (Xue et al., 2010; Yeşilbağ et al., 2017; Deng et al., 2020; Chen et al., 2021; Yang et al., 2023).

Yaks mainly inhabit the high-altitude regions of the Qinghai-Tibet Plateau and its surrounding areas. In China, yaks are distributed across the provinces and autonomous regions of Qinghai, Tibet, Sichuan, Gansu, Xinjiang, and Yunnan, with an amount of approximately 16.36 million that accounts for over 94% of the global yak population. Qinghai Province maintains China’s largest yak population at 6.15 million (37.57% of national total) (Han and Liang, 2024). Previous studies have demonstrated a wide seroprevalence range of BVDV (44.86-100%) among yak populations across different regions of Qinghai (Gao et al., 2013), with infections involving the BVDV-1b, BVDV-1d, and BVDV-1q subgenotypes (Gong et al., 2014). However, the current epidemiological status in yaks across broad geographic areas of Qinghai remains poorly characterized, which prompt our comprehensive serological and molecular epidemiological investigation of BVDV in local yak populations, with the aim of providing critical data for effective prevention and control strategies.

## 2 Materials and methods

### 2.1 Collection of serum and blood samples

From April 2024 to May 2025, a total of 1,370 yaks (aged ≥1 year) exhibiting respiratory symptoms or diarrhea but with no history of BVD vaccination were selected for this study. The animals were sampled from 19 grazing herds (total population: 15726) across eight administrative regions including Yushu, Guoluo, Huangnan, Hainan, Haidong, Haixi, Haibei, and Xining in Qinghai Province; and the sampling size exceeded 5% of the population, with additional samples collected from selected herds based on owners’ voluntary participation in the surveillance program (**Table 1**; **Supplementary Figure 1**). Non-anticoagulated whole blood samples were collected by local veterinary practitioners. The serum were isolated via centrifugation at 4, 000 rpm for 10 min and transported to the laboratory on ice. The serum samples were preserved at -80 °C until detection.

**Table 1:** Summary of sera sample and prevalence of BVDV in 19 yak herds in Qinghai province.

Administrative regions	Herd number	Numbers of samples	Population size	Antibody prevalence	Antigen prevalence	Regional antigen prevalence
Yushu	No.1	92	1800	61.96% (57/92)	2.17% (2/92)	1.54% (2/130)
	No.2	38	410	65.79% (25/38)	0.00% (0/38)	
Guoluo	No.3	198	1700	90.40% (179/198)	4.04% (8/198)	3.36% (8/238)
	No.4	40	520	62.50% (25/40)	0.00% (0/40)	
Huangnan	No.5	52	590	98.07% (51/52)	9.62% (5/52)	4.90% (5/102)
	No.6	50	700	88.00% (44/50)	0.00% (0/50)	
Hainan	No.7	66	560	90.91% (60/66)	1.52% (1/66)	1.52% (1/66)
Haidong	No.8	50	560	96.00% (48/50)	2.00% (1/50)	1.28% (1/78)
	No.9	28	340	82.14% (23/28)	0.00% (0/28)	
Haixi	No.10	93	1250	84.95% (79/93)	1.08% (1/93)	0.34% (1/293)
	No.11	50	850	12.00% (6/50)	0.00% (0/50)	
	No.12	150	2900	96.67% (145/150)	0.00% (0/150)	
Haibei	No.13	87	720	88.51% (77/87)	8.05% (7/87)	4.23% (9/213)
	No.14	60	670	91.67% (55/60)	1.67% (1/60)	
	No.15	25	400	88.00% (22/25)	4.00% (1/25)	
	No.16	41	550	90.24% (37/41)	0.00% (0/41)	
Xining	No.17	94	280	90.43% (85/94)	1.06% (1/94)	2.40% (6/250)
	No.18	73	366	93.15% (68/73)	6.85% (5/73)	
	No.19	83	560	86.75% (72/83)	0.00% (0/83)	
Total	19	1370	15726	84.52% (1158/1370)	2.41% (33/1370)	

### 2.2 BVDV antibody ELISA

The serum samples were detected of antibodies against the BVDV NSP2–3 protein by using ID Screen^®^ BVD p80 Antibody Competition Kit (IDvet Innovative Diagnostics, France) according to the user manual. Samples with competition percentage (OD_sample_/OD_negative_%) less than 40% was classified as positive.

### 2.3 BVDV antigen ELISA

All blood samples were examined for the BVDV NSP2–3 protein via ID Screen^®^ BVD p80 Antigen Capture Kit (IDvet Innovative Diagnostics, France) based on the manufacturer’s instructions. The ratio of the mean values of ((OD_sample_ - OD_negative control_)/(OD_positive control_ - OD_negative control_)) higher than 50% were considered as positive.

### 2.4 One step RT-PCR assays

The RNA was extracted from 400 μL of antigen positive serum samples using Quick-RNA/DNA Viral Kit (Zymo Research, USA) according to the manufacturer’s instructions. The viral 5-untranslated region (5’-UTR) was detected by RT-PCR using primers F (5’-CTAGCCATGCCCTTAGTAGGACTA-3’) and R (5’-CAACTCCATGTGCCATGTACAGCA-3’) (Mahony et al., 2005). The RT-PCR reaction was performed using the PrimeScript One Step RT-PCR Kit Ver.2 (Dye Plus) (TaKaRa, Dalian, China), with 50°C for 30 min, followed by 35 cycles at 94°C for 30 s, 55°C for 30 s, 72°C for 30 s, and a final extension at 72°C for 10 min. The PCR products were checked by electrophoresis on a 1% agarose gel. In order to confirm the detection result and subsequent sequence analysis based on the 5’ UTR, additional RT-PCR reactions for the N^pro^ coding region were performed with selected RNA samples, using the primers BD1 (5’-TCTCTGCTGTACATGGCACATG-3’) and BD2 (5’- TTGTTRTGGTACARRCCGTC -3’) (Vilcek et al., 1997). The reaction was carried out with identical RT-PCR reagents and a modified protocol (72°C extension for 1 min per cycle).

### 2.5 Phylogenetic analysis

The PCR products were recovered by using the Zymoclean Gel DNA Recovery Kit (Zymo Research, USA) and cloned into pMD19-T vector (TaKaRa, Dalian, China) for DNA sequencing. The obtained sequences were analyzed using NCBI BLAST program. To investigate the genetic diversity and evolutionary relationships of the identified BVDV strains, the assembled 5’-UTR and N^pro^ coding sequences were aligned against the reference NADL strain sequences (245 bp for 5’-UTR, genome positions 130-347; 385 bp for N^pro,^ genome positions 386-770) using MAFFT v7.505 (Katoh and Standley, 2013). Phylogenetic trees were conducted by the MEGA software (version 12) using the neighbor-joining (NJ) method and bootstrap analysis (n = 1,000).

## 3 Results

### 3.1 Seroprevalence of BVDV antibodies

The antibody ELISA results showed an overall BVDV antibody seroprevalence of 84.52% (1158/1370) in Qinghai yaks, with variations among herds and regions (**Table 1**). Two herds (No.1 and No.2) in Yushu exhibited seropositivity rates of 61.96% and 65.79% respectively. Yaks in Guoluo demonstrated antibody prevalence of 90.40% in one herd (Herd No.3) and 62.50% in another (Herd No.4). Except for low-prevalence in one herd (Herd No.11) in Haixi (12.00%), the BVDV antibody seroprevalence of other surveyed herds were 98.07% and 88.00% respectively in two herds (No.5 and No.6) in Huangnan, 90.91% in Hainan (Herd No.7), 96.00% and 82.14% in two herds (No.9 and No.10) in Haidong, 84.95% in one herd (No.10) and 96.67% in another (No.12) in Haixi, 88.51% (herd No.13), 91.67% (herd No.14), 88.00% (herd No.15), and 90.24% (herd No.16) in Haibei, as well as 90.43% (herd No.17), 93.15% (herd No.18) and 86.75% (herd No.19) in Xining (**Table 1**; **Supplementary Figure 1**). Since these animals were not immunized with BVD vaccine, the high antibody seroprevalence are more likely to indicates widespread transmission of BVDV among the Qinghai yak population.

### 3.2 Prevalence of BVDV antigen

The viral antigen detection results revealed a relatively high proportion of BVDV antigen-positive herds in Haibei and Xining, with herd-level prevalence of 75% (3/4) and 66.67% (2/3), respectively. This was followed by herd-level prevalences in Yushu (50%), Guoluo (50%), Huangnan (50%), Haidong (50%), and Haixi (33.33%) (**Table 1**). Additionally, one yak from the single selected herd in Hainan also tested positive for BVDV antigen, albeit at a low antigen prevalence of 1.52% for the herd (**Table 1**). It will be necessary to conduct more extensive herd testing to more accurately evaluate the antigen prevalence of BVDV in Hainan. The highest BVDV antigen prevalence (9.62%, 5/52) was observed in one herd (No.5) in Huangnan, followed by herd No. 13 in Haibei (8.05%, 7/87) and No. 18 in Xining (6.85%, 5/73). Other herds with relatively high BVDV antigen prevalence included one herd (No.3) in Guoluo (4.04%, 8/198) and one (No.15) in Haibei (4.00%, 1/25). Comparatively, the prevalence was lower in some herds of Yushu (No.1, 2.17%, 2/92), Haidong (No. 8, 2.00%, 1/50), Hainan (No.7, 1.52%, 1/66), Haixi (No.10, 1.08%, 1/93), and Xining (No.17, 1.06%, 1/94) (**Supplementary Figure 1**; **Table 1**).

As shown in supplementary **Figure 1** and **Table 1**, the region level prevalence of BVDV antigen in yaks was observed in Huangnan (4.90%, 5/102) and Haibei (4.23%, 9/213), which likely resulted from higher breeding density or frequent animal movement. Intermediate viral antigen prevalence levels were exhibited in yaks form Guoluo (3.36%, 8/238) and Xining (2.40%, 6/250), but the inter-herd differences should not be neglected. Notably, the prevalence of viral antigen in Haixi was lowest the 0.34% (1/293), which may be attributed to better biosecurity measures or lower farm density.

**Figure 1:**
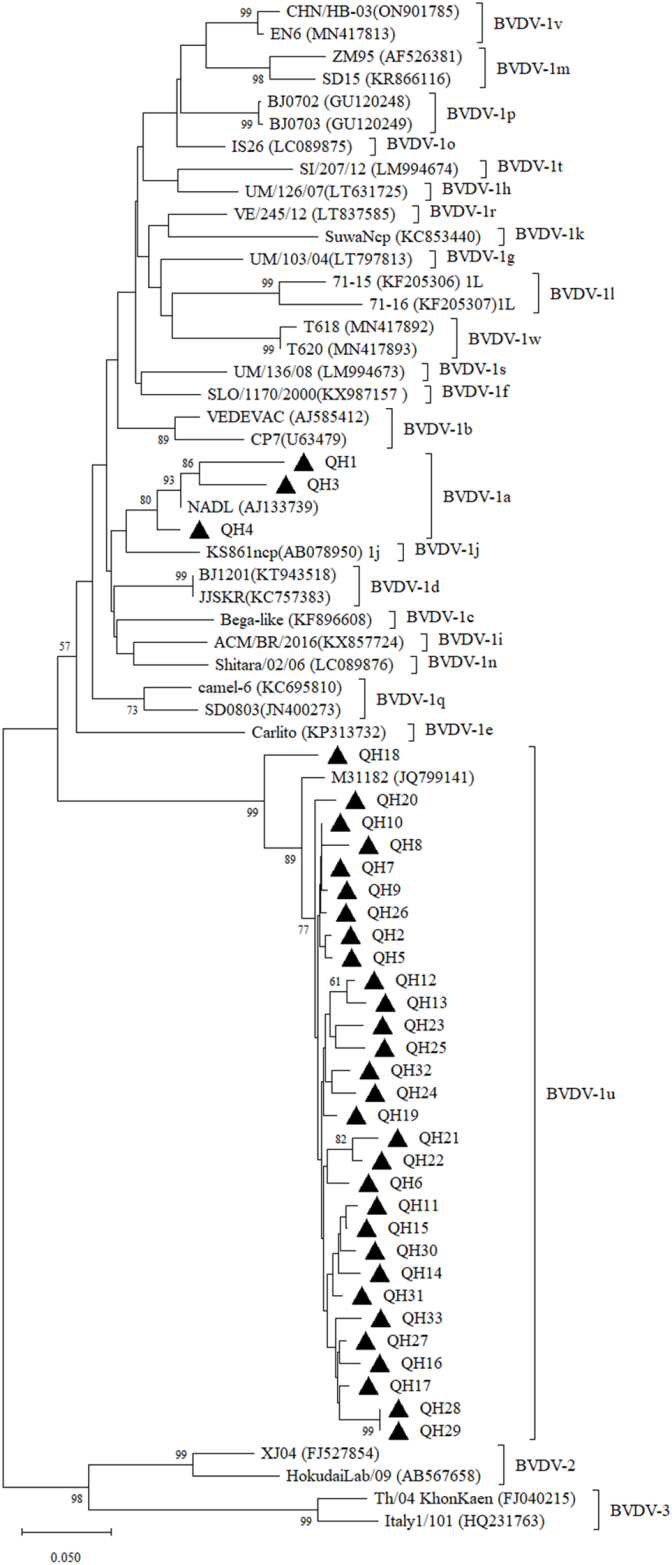
Neighbor-joining phylogeny of BVDV isolates based on the 5’-UTR sequences (245 bp). The phylogenetic tree was constructed using the neighbor-joining method implemented in MEGA version 12. Bootstrap values are percentage of 1000 replicates and are shown below the branches. Bar indicates substitutions per site. Accession numbers representative subgenotypes are listed in the brackets after the isolate names.

### 3.3 Sequencing and phylogenetic analysis

We further validated the virus in antigen-positive samples using RT-PCR. The results showed that the 5’-UTR fragment was successfully amplified in all 33 RNA samples prepared from antigen-positive sera. The obtained 5’-UTR sequences were submitted to GenBank under accession number PV806868-PV806900. The 5’-UTR sequences were aligned and a sequence identity matrix was generated, demonstrating that the 5’-UTR of the Qinghai strains shared nucleotide identity ranged from 74.90-100% (**Supplementary Figure 2**). Phylogenetic analysis revealed that these 33 samples belonged to the BVDV-1a (n=3) and BVDV-1u (n=30) subgenotypes (**Figure 1**). Among them, BVDV-1a was detected in yaks from two regions including Guoluo and Yushu, while BVDV-1u was distributed in yaks from all the eight administrative regions in Qinghai Province. The 5’-UTR sequence of BVDV-1a strains (QH1, QH3, QH4) shared 92.10-92.50% nucleotide identity, while BVDV-1u strains (QH2, QH5 to QH33) displayed much wider variability, sharing nucleotide identity ranging from 90.10% to 100%. These 5’-UTR sequences of Qinghai BVDV-1u strains shared 93.70-97.60% nucleotide identity with an BVDV isolate M31182 (GenBank accession number: JQ799141) originated from yaks in neighboring Sichuan Province, which was identified as BVDV-1u in a previous study (Deng et al., 2020). Notably, the 5’-UTR of QH18 exhibited significant divergence from other BVDV-1u strains, sharing only 90.10-93.70% nucleotide identity, whereas the 5’-UTR sequences of the remaining BVDV-1u strains showed much higher intra-group similarity (93.7-100%). The phylogenetic tree based on the 5’-UTR demonstrated that QH18 occupied a distinct phylogenetic position, clearly separated from other BVDV-1u strains (**Figure 1**).

**Figure 2:**
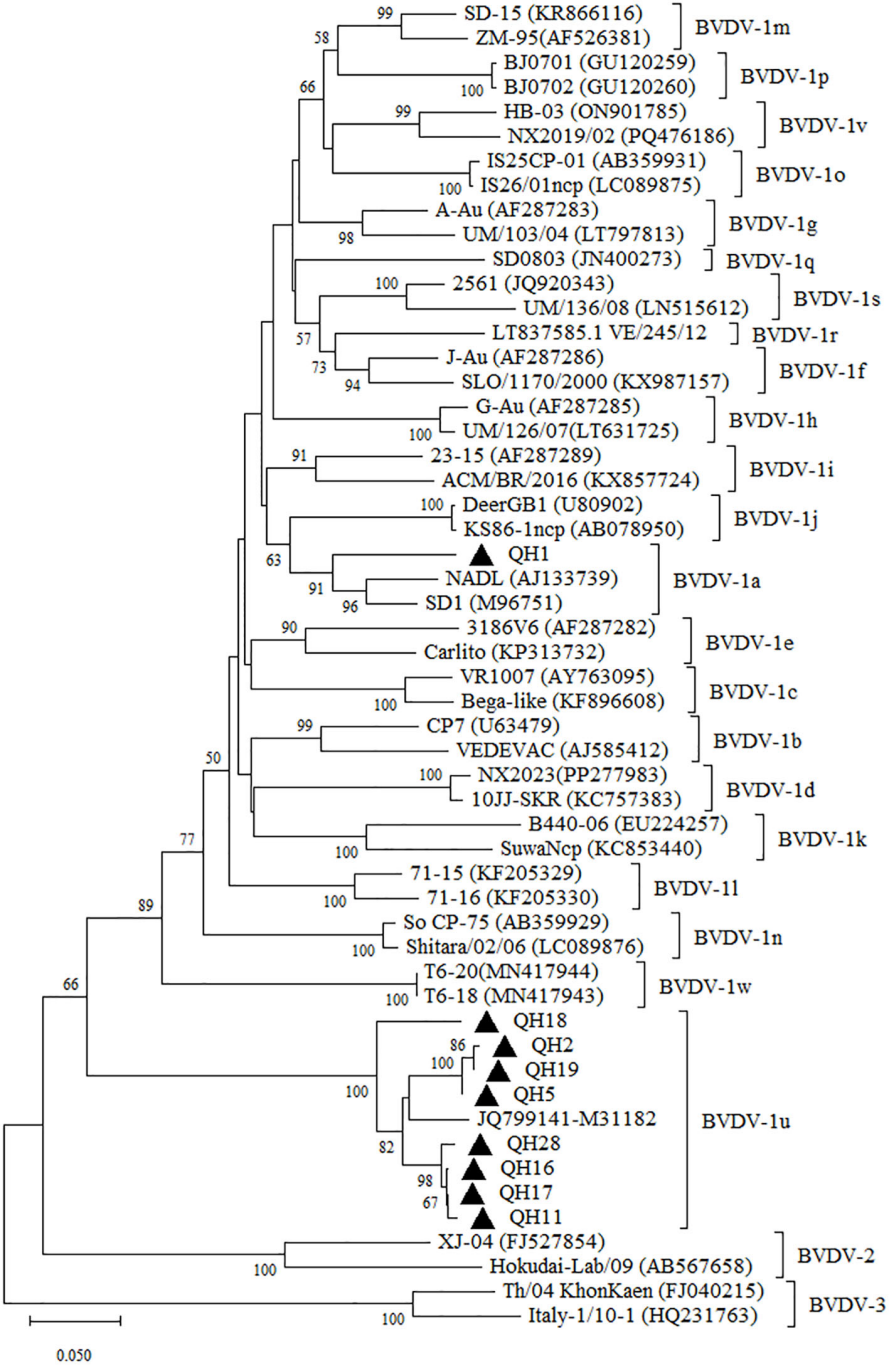
Neighbor-joining phylogeny of BVDV isolates based on N^pro^ coding region (385 bp). The phylogenetic tree was constructed using the neighbor-joining method implemented in MEGA version 12. Bootstrap values are percentage of 1000 replicates and are shown below the branches. Bar indicates substitutions per site. Accession numbers representative subgenotypes are listed in the brackets after the isolated names.

To further validate the subgenotyping analysis based on the 5’-UTR, we conducted complementary sequencing and phylogenetic analysis of the N^pro^ gene from representative regional samples. The obtained N^pro^ gene coding sequences were submitted to GenBank under accession number and PV806901-PV806909. The QH1 strain had 95.20% and 93.0% nucleotide identity with the N^pro^ gene sequences of the Chinese BVDV-1a strains BJ-2013 (GenBank No. MH490942) and HLJ-2018 (GenBank No. PV168122). Compared with the M31182 isolate, the N^pro^ gene of Qinghai BVDV-1u strains shared 89.00~94.00% nucleotide identity, with QH18 displaying the lowest homology (89.00%). The phylogenetic analysis of the N^pro^ gene yielded results consistent with those based on the 5’UTR region, with QH18 similarly positioned on a distant evolutionary branch (**Figure 2**). These findings suggest the potential existence of undetected genetically divergent BVDV-1u strains in yak populations in Qinghai, emphasizing the critical need for further molecular epidemiological studies to enable more detailed characterization of BVDV in Qinghai yaks.

## 4 Discussion

Since the first report in 1946, BVD continues to be one of the primary causes of significant economic and production losses in the cattle industry (Newcomer, 2021). BVDV infects not only cattle but also a wide range of artiodactyls, including swine, domestic ruminants such as sheep and goats, camelids like alpacas and camels, and wildlife such as cervids, antelope, wild goats and sheep, and American bison (*Bison bison*), and infections in heterologous hosts may result in mild or similar clinical signs to BVDV infections in cattle and generate persistently infected carriers, posing risks as viral reservoirs (Passler and Walz, 2010; Van Campen and Rhyan, 2010; Nelson et al., 2016; Hause et al., 2021). In this study, we conducted a comprehensive investigation of (BVDV) in yak populations in the most important province for yak farming in China utilizing serological antibody detection, viral antigen profiling, and molecular genotyping. Prior to our investigation, BVDV infection in yaks had indeed attracted considerable attention. The initial detection of BVDV in China occurred during the 1980s, with near-simultaneous identifications in both cattle and yak (Li et al., 1983; Liu, 1984). However, the majority of studies of BVD in yak were published in Chinese journals, significantly limiting their accessibility to the international readers. A recently systematic review and meta-analysis illustrated that BVDV in yaks between 1987 and 2019 in China had a variable prevalence of 24.4%-67.5% and viral antigen-positive rate of 13.8% (Diao et al., 2020). In our study, the overall observed seroprevalence of BVD (84.52%) was comparable with the 72.14% prevalence reported in a previous study (Gao et al., 2013). Some discrepancies can be attributed to our targeted selection of yaks exhibiting certain clinical symptoms rather than random screening. Additionally, since classical swine fever (CSF)vaccines are sometimes used for prevention yaks from BVD in China (Ji et al., 1995; Liu et al., 2013), which may interfere with antibody detection. Therefore, we specifically selected yak herds that had not been vaccinated against either BVD or CSF vaccines for our seroprevalence study to accurately reflect the true infection risk within the population.

Notably, one herd in Haixi (No. 11) showed relatively low seroprevalence at the animal level, suggesting a reduced exposure to circulating virus. In cattle, BVDV transmission primarily relies on high viral shedding from persistently infected animals, with transiently infected animals playing a secondary role due to their lower and shorter-term viral excretion (Evans et al., 2019), and a similar dynamic occurs in non-bovine ruminant species (Passler and Walz, 2010; Van Campen and Rhyan, 2010; Nelson et al., 2016; Hause et al., 2021). The observation of seroprevalence in the herd in Haixi correlated with local BVDV antigen testing results, which indicated a markedly lower prevalence of BVDV antigen in this region compared with other study areas. Due to the free-range grazing practices of yaks, the epidemiological characteristics of diseases may differ from those in intensive indoor feeding systems. Previous studies showed yaks are prone to co-infections with bovine rotavirus, bovine enterovirus, bovine astrovirus, bovine coronavirus, bovine parainfluenza virus, *Pasteurella multocida* and *Escherichia coli* alongside BVDV (Yuan et al., 2015; Yang et al., 2019). Consequently, in clinically affected yak populations exhibiting low BVDV prevalence, potential co-infecting pathogens should be systematically evaluated in future studies.

The identification of BVDV antigen provided direct and definitive evidence for confirming the presence of progressive BVDV infection in eight yak herds. Compared with provincial survey data from a decade ago (Fu et al., 2013), the results obtained in our study were generally consistent while exhibiting certain discrepancies. The yaks in Huangnan Prefecture continued to exhibit the highest BVD prevalence (2.21% vs. 4.90%), while Haixi Prefecture maintains the lowest prevalence (0.43% vs. 0.34%), suggesting that stable factors such as climatic characteristics, livestock production practices may influence BVD prevalence in these two regions. Notably, increases in BVDV antigen prevalence in yaks in Haibei (0.43% to 4.23%) and Guoluo Prefecture (0.43% to 3.36%) were observed. Although we cannot rule out biases caused by different sampling methods and reagent sensitivities, the detection of antigens in the yak population indicates that the monitoring and prevention of BVDV in yaks must not be overlooked. Moreover, the detection of BVDV antigen in yaks from all regions suggests that all newly introduced yaks should be quarantined to confirm their BVDV-negative status before herd introduction.

The conserved 5’-UTR and the N^pro^ coding region are widely used for genotyping phylogenetic analysis for BVDV, providing critical insights into the genetic relatedness, evolutionary divergence, and possible origins of the circulating BVDV strains. Consistent with the national BVDV epidemiological pattern (Xiao et al., 2025), our study confirmed BVDV-1 as the predominant genotype, while revealing unique regional characteristics. Notably, although BVDV-1m has been reported in Qinghai dairy cattle (Xiao et al., 2025), this subgenotype remains rarely documented in yaks. Alongside earlier reports of BVDV-1b, -1d, and -1q (Gong et al., 2014) and the recent identification of BVDV-1a in Haibei (Lin et al., 2025), our findings reveal that BVDV-1u has emerged as the dominant subgenotype across Qinghai yaks, while BVDV-1a remains at low prevalence. In comparison with previous studies, our findings revealed a shift in subgenotypes in yaks in Qinghai Province, suggesting potential ongoing viral evolution or host adaptation. Although relatively few Qinghai BVDV-1a strains were obtained in neighboring Yushu and Guoluo prefectures, the sequences among these strains were not identical. Therefore, the polymorphism of the Qinghai 1a subtype cannot be overlooked, and more sequences need to be acquired in the future for further verification. Furthermore, the genetic analysis of BVDV-1u sequences obtained in this study revealed significant polymorphism within this subgenotype, with QH18 exhibiting notable divergence from other strains, possibly resulting from unique selective pressures due to host adaptation or geographic isolation. The QH18 strain displays the lowest nucleotide similarity among BVDV-1u strains, with only 90.1% identity in the 5’UTR and 90.2–90.8% in the N^pro^ gene compared to other Qinghai 1u isolates, followed by the Sichuan yak strain (M31182). Such divergence could have implications for viral pathogenicity and vaccine efficacy. Despite its current classification within subgenotype 1u, the considerable divergence observed in QH18 suggests that ongoing evolution may lead to the emergence of a distinct subgenotype, emphasizing the need for sustained surveillance. Given the vast territory of Qinghai and the free-range grazing practices of yaks across wide areas, we were unable to conduct systematic monitoring of persistent infection status BVDV in yaks. However, a recent study in Tibet revealed a persistent BVDV infection rate of 1.55% in yaks through antigen testing of paired sera samples collected at 3-week intervals (Luo et al., 2023). On the Qinghai-Tibet Plateau, the yak population substantially exceeds that of domesticated cattle, and persistent infection of BVDV in yak may suggest that the yak can sustain BVDV transmission cycles, but whether they can act as true reservoirs remains unclear. The limited BVDV data resulted in inconsistent subgenotype comparation between yak and cattle, highlighting the need to investigate potential epidemiological links between yaks under free-range grazing systems and intensively managed domestic cattle in Qinghai, which would be a crucial direction for future molecular epidemiological research on BVDV.

## 5 Conclusion

In conclusion, this study systematically elucidates the epidemiological characteristics and predominant subgenotypes of BVDV in yaks in Qinghai Province, highlighting the need for investigation of the transmission dynamics of BVDV under the unique ecological conditions and the development of effective intervention measures for preventing and controlling BVD for yak.

## Data Availability

The datasets presented in this study can be found in online repositories. The names of the repository/repositories and accession number(s) can be found in the article/**Supplementary Material**.

## Supplementary material

Supplementary Figure 1 The geographic distribution of BVDV from the nineteen herds of yaks in Qinghai Province.
