# Supplementary figures and images for: Comprehensive molecular epidemiology of BVDV in yaks (Bos gruniens) in Qinghai, China: high prevalence and dominance of BVDV-1u
Source: Front Cell Infect Microbiol. 2025 Aug 14;15:1652023. doi: 10.3389/fcimb.2025.1652023 (PMC12391088; doi:10.3389/fcimb.2025.1652023)

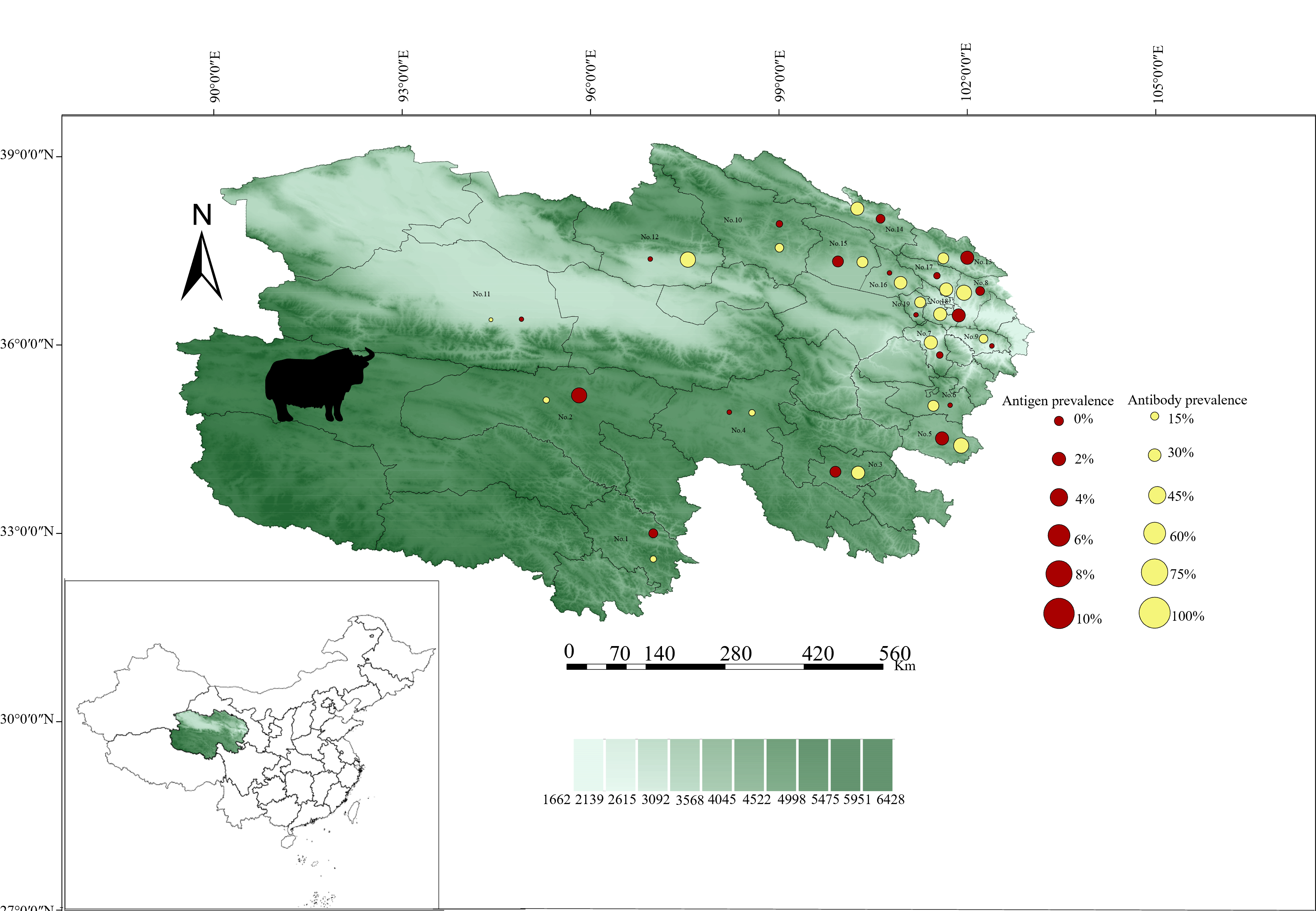

Supplement: Supplementary Figure 1 — The geographic distribution of BVDV from the nineteen herds of yaks in Qinghai Province. [file Image1.tif]

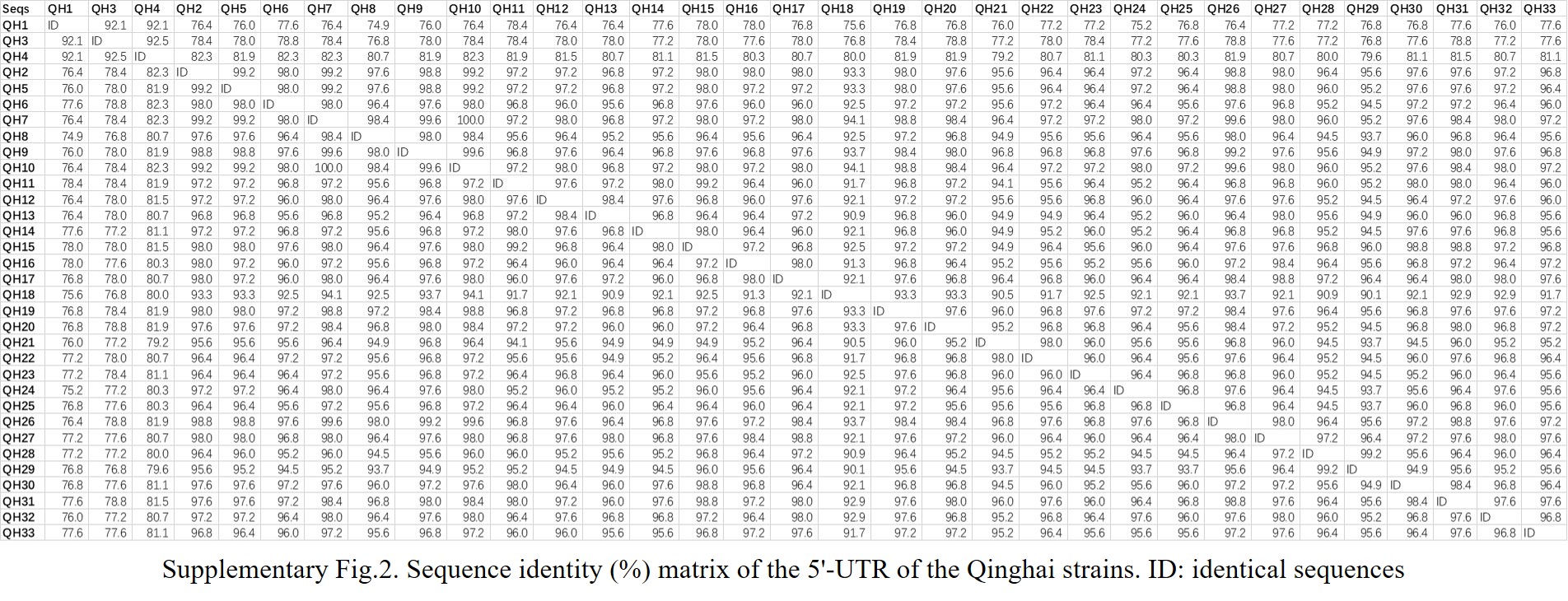

Supplement: Supplementary file 2 [file Image2.tif]
